# A novel thermostable xylanase GH10 from *Malbranchea pulchella* expressed in *Aspergillus nidulans* with potential applications in biotechnology

**DOI:** 10.1186/1754-6834-7-115

**Published:** 2014-07-29

**Authors:** Liliane FC Ribeiro, Rosymar C De Lucas, Gabriela L Vitcosque, Lucas F Ribeiro, Richard J Ward, Marcelo V Rubio, Andre RL Damásio, Fabio M Squina, Rebecca C Gregory, Paul H Walton, João A Jorge, Rolf A Prade, Marcos S Buckeridge, Maria de Lourdes TM Polizeli

**Affiliations:** Immunology and Biochemistry Department of Faculdade de Medicina de Ribeirão Preto - USP, Ribeirão Preto, SP Brazil; Chemistry Department of Faculdade de Filosofia, Ciências e Letras de Ribeirão Preto - USP, Ribeirão Preto, SP Brazil; Laboratório Nacional de Ciência e Tecnologia do Bioetanol (CTBE), Campinas, SP Brazil; Department of Chemistry, The University of York, York, UK; Biology Department of Faculdade de Filosofia, Ciências e Letras de Ribeirão Preto - USP, Av. Bandeirantes, 3900, Ribeirão Preto, SP 14040-901 Brazil; Department of Microbiology and Molecular Genetics, Oklahoma State University, Stillwater, OK USA; Institute of Biosciences, University of São Paulo, São Paulo, Brazil

**Keywords:** Xylanase, *Malbranchea*, Glycosylation, Heterologous expression, Thermostability, Biomass degradation, Sugarcane bagasse

## Abstract

**Background:**

The search for novel thermostable xylanases for industrial use has intensified in recent years, and thermophilic fungi are a promising source of useful enzymes. The present work reports the heterologous expression and biochemical characterization of a novel thermostable xylanase (GH10) from the thermophilic fungus *Malbranchea pulchella*, the influence of glycosylation on its stability, and a potential application in sugarcane bagasse hydrolysis.

**Results:**

Xylanase MpXyn10A was overexpressed in *Aspergillus nidulans* and was active against birchwood xylan, presenting an optimum activity at pH 5.8 and 80°C. MpXyn10A was 16% glycosylated and thermostable, preserving 85% activity after 24 hours at 65°C, and deglycosylation did not affect thermostability. Circular dichroism confirmed the high alpha-helical content consistent with the canonical GH10 family (β/α)_8_ barrel fold observed in molecular modeling. Primary structure analysis revealed the existence of eight cysteine residues which could be involved in four disulfide bonds, and this could explain the high thermostability of this enzyme even in the deglycosylated form. MpXyn10A showed promising results in biomass degradation, increasing the amount of reducing sugars in bagasse *in natura* and in three pretreated sugarcane bagasses.

**Conclusions:**

MpXyn10A was successfully secreted in *Aspergillus nidulans*, and a potential use for sugarcane bagasse biomass degradation was demonstrated.

## Background

Thermophilic and thermotolerant fungi are a potential source of enzymes with novel properties. Due to the fact that these organisms grow at high temperatures, their cellular constituents such as enzymes together with organized cellular structures, such as ribosomes and membranes, must also function at higher temperatures [1]. Novel thermostable enzymes that degrade plant cell walls are of interest, as they can be applied in many different industrial contexts [2]. Hemicellulose is the second major component present in the plant cell wall, and thermostable xylanases have been investigated for their potential application in biomass degradation in the pulp and paper industry, feed industry, and bioethanol production [3].

Xylanases of the GH10 family are either endo-1,4-beta-xylanases or endo-1,3-beta-xylanases (EC 3.2.1.8), which have molecular masses ≥30 kDa and low isoelectric points [4], and their crystal structure displays a (β/α)_8_ barrel architecture [5]. Catalytic activity kinetic assays and end product quantitation analyses, along with enzyme structural studies, indicate that GH10 xylanases typically present four to five substrate-binding subsites [4].

The conversion of plant lignocellulose biomass into fermentable sugars has been the focus of recent investigation, since such a technology may be used to produce sustainable biofuels and in biorefineries, in which renewable resources such as agricultural waste may be utilized for direct bioconversion into chemicals and fuels. This potential has stimulated research in the use of biocatalysts for bioenergy production [3], and in this context, thermostable enzymes have an obvious advantage as catalysts in these processes, since high temperatures often promote better enzyme penetration and cell wall disorganization [3].

*Malbranchea pulchella* is a thermophilic fungus isolated from soil and decaying vegetation. Several studies have described enzymes produced by this fungus, such as the purification and characterization of a trehalase [6], and the demonstration of high level production of thermostable cellulolytic and xylanolytic enzymes [7]. Furthermore, other species from the *Malbranchea* genus have been shown to be good enzyme producers, especially of proteases [8, 9], and the production of alpha-amylase, beta-xylosidase, chitinase, and xyloglucanase has also been reported [10–13].

Heterologous protein expression in filamentous fungi presents three main advantages when compared to the expression of a fungal enzyme in another host. First, filamentous fungi secrete proteins in large quantities which facilitate their purification and characterization. The second advantage is that most genes from fungi have introns and filamentous fungi are able to recognize and process them correctly. Finally, many fungal proteins are glycosylated, and the protein expression in another filamentous fungus results in a glycosylation pattern that is highly similar to that of the native fungal enzyme to be studied [14].

Furthermore, the filamentous fungi are able to produce and secrete considerable quantities of protein, and the main advantages of expressing a fungal enzyme in another filamentous fungus are the similar protein synthesis machinery and the glycosylation processes. This work describes the characterization of a secreted thermostable GH10 xylanase from *Malbranchea pulchella* expressed in *Aspergillus nidulans* using the pEXPYR vector. Xylanase GH10 was expressed using the signal peptide from the *M. pulchella* enzyme, from which a high amount of the thermostable enzyme was obtained.

## Results and discussion

### Cloning and expression of MpXyn10A in *Aspergillus nidulans*

**Figure 1:**
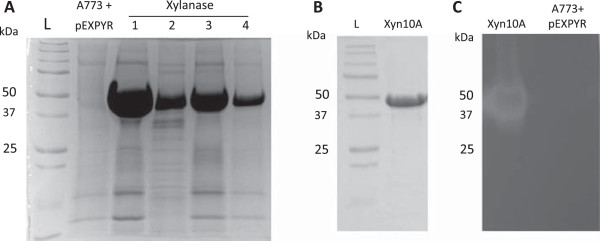
**Expression and zymogram of MpXyn10A in** ***A. nidulans.*** **(A)** SDS-PAGE analysis of the culture filtrate recovered from flask cultures; 1-4) Four different transformants of *A. nidulans* carrying the MpXyn10A ORF after 5% maltose induction for 48 h at 37°C, under static conditions. L) ladder; A773 + pEXPYR) *A. nidulans* A773 transformed with empty pEXPYR after 5% maltose induction for 48 h at 37°C, under static conditions. **(B)** SDS-PAGE of purified MpXyn10A. **(C** **)** Zymogram of the crude extract of *A. nidulans* expressing MpXyn10A (Xyn10A) and of *A. nidulans* transformed with empty pEXPYR (A773 + pEXPYR) after 5% maltose induction for 48 h at 37°C. See Methods section for further details.

A putative open reading frame (ORF) encoding *Malbranchea pulchella xyn10A* was amplified by PCR from genomic DNA with primers designed from a multiple nucleotide sequence alignment of xylanase GH10 ORFs deposited in GenBank (accession number [GenBank:KJ767674]). The sequenced PCR product was 1,339 bp long and encoded an ORF with 496 amino acid residues including a 19-amino acid N-terminal signal peptide, suggesting a mature protein of 40,747 Da. The *Mpxyn10A* protein-coding DNA sequence, including the resident signal peptide, was ligated in-frame into the expression and secretion vector pEXPYR and transformed into the high protein producing strain *Aspergillus nidulans* A773 [15]. Figure 1A shows sodium dodecylsulfate-polyacrylamide gel electrophoresis (SDS-PAGE) profiles of four independent transformants expressing and secreting *Mpxyn10A* in the host *A. nidulans*. The secreted protein band shown in Figure 1A was isolated and analyzed by LC-MS/MS (Orbitrap Thermo Scientific) and confirmed to be *M. pulchella* xylanase GH10.

### Purification and characterization of MpXyn10A

MpXyn10A was expressed at high levels (Figure 1A) and purified with size exclusion chromatography (Biogel P-60, Figure 1B). A zymogram analysis showing the xylanase activity was performed in parallel to SDS-PAGE for the confirmation of the heterologous expression and secretion of xylanases (Figure 1C) corroborating with LC-MS/MS analysis. This activity band is not observed in the crude extract of the host fungus transformed with the empty vector, confirming that the observed protein is the overexpressed MpXyn10A.

**Figure 2:**
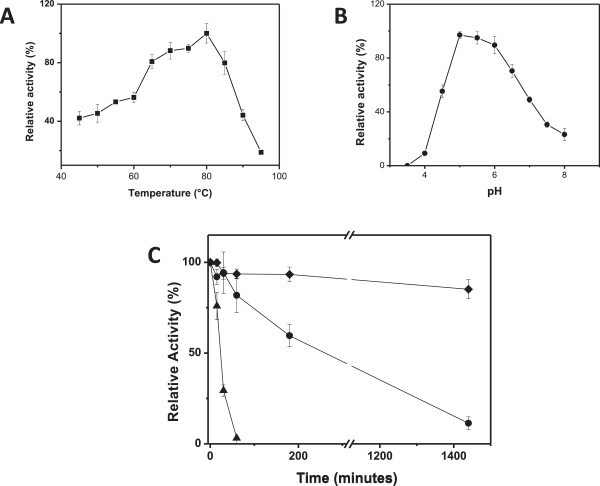
**Effect of temperature and pH and the thermal stability of MpXyn10A. (A)** Effect of temperature on xylanase activity. The activity was measured on incubating xylan birchwood (substrate) in 50 mM sodium acetate buffer, pH 5.0, at the tested temperatures for 10 min. **(B)** Effect of pH on xylanase activity (from 3.5 to 8.0). The activity was measured upon incubating xylan birchwood (substrate) in 100 mM sodium citrate/phosphate buffer at the different pH values tested at 80°C for 10 min. **(C)** Xylanase activity thermostability. Black triangles 80°C; black circles 70°C; black diamonds 65°C. The activity was measured upon incubating xylan birchwood (substrate) in 50 mM sodium acetate buffer, pH 5.5, at 80°C for 10 min. In all experiments the detection of reducing sugars released was conducted as previously described by Miller [33].

The temperature and pH optima, together with the thermal stability of the purified enzyme, were determined (Figure 2A, B, and C). MpXyn10A presented the highest catalytic activity at 80°C and pH 5.5 (Figure 2A and B, respectively). Similar results were also obtained for *Thermoascus aurantiacus* [16] and *Penicillium funiculosum* xylanases [17], which presented optimum activities at 80°C and at pH 5.1; 4.0 to 5.5, respectively. The beta-xylosidase activity of the purified recombinant enzyme was also tested, but no activity was detected using a pNP-xylopyranoside substrate. Previous work describing the partial characterization of a xylanase purified from *Malbranchea pulchella* culture supernatants reported an optimum catalytic temperature of 70°C and optimum pH in the range of 6.0 to 6.5 [18]. The present work reports for the first time the expression, purification and characterization of a xylanase GH10 from *Malbranchea pulchella* (MpXyn10A). MpXyn10A retained about 85% activity after 24 h incubation at 65°C (Figure 2C); however, at 70°C the enzyme lost 20% of its activity after 1 h. The MpXyn10A exhibited a higher thermal stability in comparison to the GH10 xylanase from *Sporotrichum thermophile* [19], which was stable for 1 h at 50°C, but presented less than 80% activity after 1 h at 60°C. The K_M_, V_max_, k_cat_, and k_cat_/K_M_ of the recombinant MpXyn10A against birchwood xylan were 4.6 mg.mL^-1^, 82 μmol.min^-1^.mg^-1^, 748 s^-1^, and 162.6 mL.mg^-1^.s^-1^, respectively (see Table 1).

**Table 1:** **Kinetic parameters of MpXyn10A obtained using birchwood xylan as substrate in 50 mM sodium acetate buffer, pH 5.5, after incubation at 80°C for 10 min**

V _max_ (μmol.min ^-1^.mg ^-1^)	K _M_ (mg.mL ^-1^)	k _cat_ (s ^-1^)	K _cat_/K _M_ (mL.mg ^-1^.s ^-1^)
82 ± 2.8	4.6 ± 0.3	748 ± 8.1	162.6 ± 1.2

The crude extract from *A. nidulans* expressing MpXyn10A was used in a comparison of xylanolytic activity with the crude extract from the wild-type fungus *Malbranchea pulchella* (see Table 2). The apparent specific activity of the *A. nidulans* crude extract containing MpXyn10A was about eightfold higher than that obtained from the culture supernatants of the *M. pulchella*, demonstrating the benefits of the overexpression of this enzyme in *A. nidulans*. The MpXyn10A therefore exhibits qualities such as high thermostability and abundant expression/secretion in the expression system used. These properties make the enzyme a promising candidate for use in industrial applications.

**Table 2:** **Xylanase activities of MpXyn10A and wild-type** ***Malbranchea pulchella*** **against birchwood and oat spelt xylan as substrates**

	MpXyn10A		***Malbranchea pulchella***	
Xylanase activity	Birchwood	Oat spelt	Birchwood	Oat spelt
U/mL	5.867 ± 0.102	6.175 ± 0.301	2.985 ± 0.051	3.946 ± 0.030
U/μg of protein	838.3 ± 7.1	882.1 ± 10.1	99.5 ± 3.2	131.5 ± 1.4

This experiment was conducted by incubating the MpXyn10A with 1% xylan in 50 mM sodium acetate buffer, pH 5.5, at 80°C for 10 min. The amount of reducing sugars released was quantified by the Miller method [33].

### Deglycosylation reduced MpXyn10A specific activity

**Figure 3:**
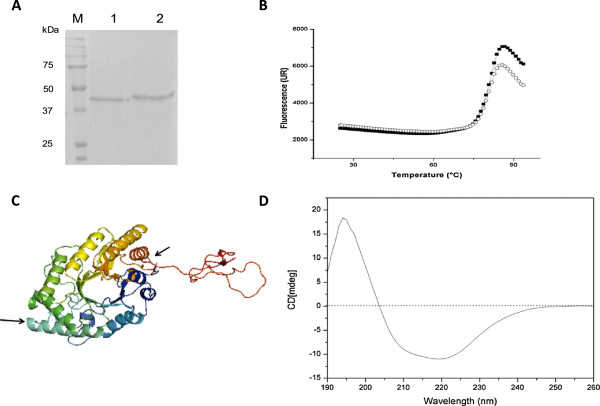
**The effect of glycosylation on MpXyn10A and structural analyses. (A)** Analyses of MpXyn10A glycosylation by SDS-PAGE. L - ladder; 1 - MpXyn10A treated with endoglycosidase H (Endo H) and purified; 2 - untreated MpXyn10A. **(B)** Differential scanning fluorimetry of glycosylated (solid squares) and deglycosylated (open circles) MpXyn10A. The melting temperature was estimated at 80.34°C for the glycosylated enzyme, with R^2^ = 0.9956, and 80.13°C for the deglycosylated enzyme, with R^2^ = 0.9975. **(C)** Three-dimensional structure of MpXyn10A generated by molecular modeling. Black arrows indicate the predicted glycosylation sites. **(D)** Far-UV circular dichroism spectrum of MpXyn10A. See Methods section for further experimental details.

The estimated molecular mass of the glycosylated MpXyn10A was approximately 49 kDa, and after deglycosylation with endoglycosidase H (Figure 3A), the enzyme presented a reduced molecular mass as estimated by SDS-PAGE. Although an accurate estimate of the molecular mass of the enzyme by SDS-PAGE was not possible due to the proximity of the bands on the gel, the ESI-MS analyses of the whole protein showed a mass of 41.6 kDa after deglycosylation. This value is close to that predicted for the molecular mass of the mature protein (40.8 kDa) calculated from the translated nucleotide sequence. The difference between the expected and the observed masses may be due to the fact that this enzyme presents two N- and nine O-glycosylation predicted sites estimated by NetNGlyc1.0 and NetOGlyc3.1, respectively (data not shown).

In order to evaluate the effect of glycosylation on thermostability of the MpXyn10A, differential scanning fluorimetry (DSF) was performed to determine the melting temperature (Tm) before and after deglycosylation. There was no difference between the temperatures of the glycosylated and deglycosylated enzyme (Figure 3B, Tm = 80.34 and 80.13°C, respectively), indicating that in this case glycosylation does not affect the thermal stability. Kinetic measurements of the glycosylated and deglycosylated enzyme revealed that the xylanase specific activity was reduced by 12% after deglycosylation (data not shown).

Family GH10 xylanases from many thermophilic fungi have been produced, isolated, and, characterized. However, the heterologous xylanase expressed in this work has been shown to be highly thermostable, retaining 85% of its activity after 24 h of incubation at 65°C. Although this enzyme presents about 16% (w/w) glycosylation, deglycosylation did not alter its stability.

### Molecular modeling

A *Fusarium oxysporum* GH10 xylanase, with Protein Data Bank (PDB) code 3U7B [5], sharing 56% identity with MpXyn10A, was used as a structural model for the catalytic domain. MpXyn10A also has a cellulose-binding domain, and for this the cellulose-binding module (CBM1) of the endoglucanase I from *Trichoderma reesei* (PDB code 4BMF [20]) was used as a template. The templates thus identified, GH10 XYLANASE from *Fusarium oxysporum* (PDB code 3U7B [5]) and a cellulose-binding domain (CBD) of endoglucanase I from *Trichoderma reesei* (PDB code 4BMF [11]) were used for building a structural model of the XYN10A by comparative modeling techniques with the program MODELLER9v12 [21].

Three-dimensional modeling of the MpXyn10A was performed using the program MODELLER, and the structure generated (Figure 3C) has a (β/α)_8_ barrel fold, which is characteristic of the family GH10 xylanases. Moreover, this enzyme has a cellulose- binding domain (CBM1) shown in red in the structure. The model structure presented 38% alpha-helix and 13% beta-sheet, and the location of the putative N-glycosylation sites (as predicted by NetNGlyc1.0) are indicated by the black arrows in Figure 3C.

The far-UV CD spectrum of purified MpXyn10A at pH 7.0 presents a maximum at 194 nm and a minimum at 220 nm, which are spectral features typical of proteins rich in alpha-helices (Figure 3D). The percentage of secondary structure elements in the protein was estimated using the results from far-UV CD, which showed that the entire protein presents 37.2% alpha-helix and 18.8% beta-sheet. This is typical of proteins containing a mixture of alpha-helix and beta-sheet, and is in agreement with the α/β content observed in the modeled three-dimensional structure of the enzyme and is also consistent with the far-UV CD spectra of other GH10 enzymes [22].

Analysis of the three-dimensional model structure revealed that all eight cysteine residues in the MpXyn10A are favorably located and in correct orientations to form disulfide bridges. The catalytic domain contains four cysteine residues that form two disulfide bridges (Cys83-Cys125 and Cys274-Cys268), and the CBM1 domain contains four cysteine residues that form another two disulfide bridges (Cys351-Cys368 and Cys362-Cys378). A possible reason for the observed high thermal stability of the deglycosylated MpXyn10A may be due to the formation of these four disulfide bonds.

### Binding assay with MpXyn10A

The CBM1 domain in the MpXyn10A may provide an anchor for the enzyme on the cellulose fiber, with the consequence that the enzyme becomes closely associated with its substrate [23]. To understand the role of the CBM1 domain, the binding of both the glycosylated and deglycosylated forms of the enzyme to cellulose (Avicel) and oat spelt xylan was investigated. The xylanolytic activity of the enzyme was measured for both the MpXyn10A bound to the polysaccharide and that existing free in the supernatant (the results are shown in Table 3). In the presence of Avicel, the xylanolytic activity was mainly detected bound to the polysaccharide, while the activity was detected mostly in the supernatant against oat spelt xylan. These results suggest the importance of the CBM1 for the activity of the MpXyn10A against natural substrates.

**Table 3:** **Test of the CBM1 functionality**

	Supernatant				Bound to the polysaccharide			
	Glycosylated		Deglycosylated		Glycosylated		Deglycosylated	
	Avicel	Oat spelt	Avicel	Oat spelt	Avicel	Oat spelt	Avicel	Oat spelt
Relative activity (%)	21.5 ± 2.3	88.9 ± 3.9	27.2 ± 2.1	85.2 ± 4.2	78.5 ± 3.3	11.1 ± 2.8	72.8 ± 5.1	14.8 ± 3.1

Glycosylated or deglycosylated MpXyn10A was incubated with Avicel or oat spelt xylan for 30 min at 4°C under agitation in 50 mM sodium acetate buffer, pH 5.5. The supernatant was separated from the polysaccharide, and the xylanolytic activity of each fraction was measured by incubation with 0.5% birchwood xylan at 70°C for 10 min. The amount of reducing sugars released was measured by the Miller method [33].

### Capillary electrophoresis analysis of MpXyn10A hydrolysis profile

**Figure 4:**
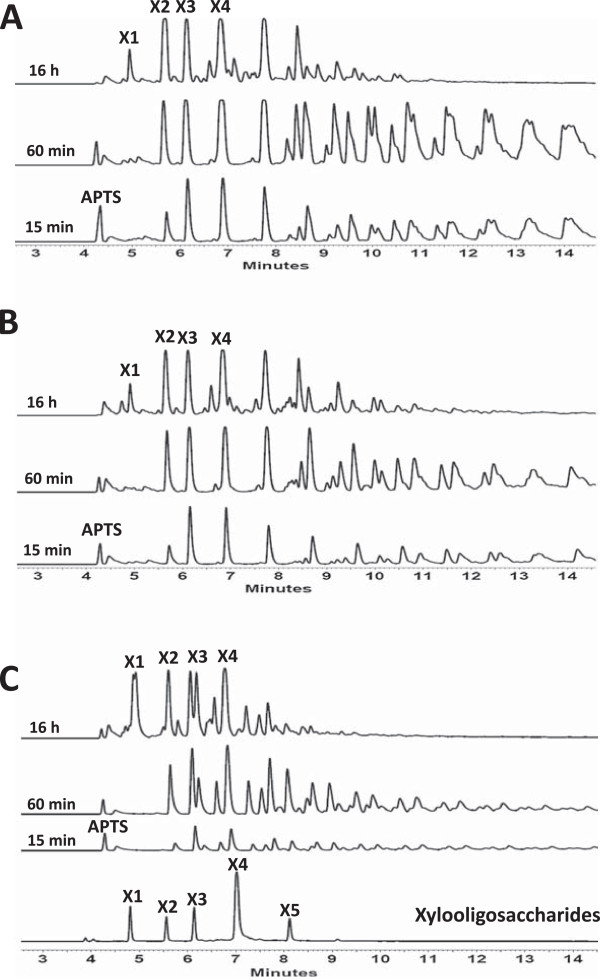
**Capillary electrophoresis analysis of xylan hydrolysis products by MpXyn10A. (A)** Products released after incubation of 1% birchwood xylan with MpXyn10A. **(B)** Products released after incubation of 1% beechwood xylan with MpXyn10A. **(C)** Products released after incubation of 1% oat spelt xylan with MpXyn10A. The assays were conducted for 15 min, 60 min, and 16 h incubation at 80°C in 50 mM sodium acetate buffer, pH 5.5.

In order to characterize the MpXyn10A hydrolysis product profile, the enzyme was incubated with xylans from birchwood, beechwood, and oat spelt (Figure 4). The hydrolysis profiles using birchwood xylan were very similar to those of beechwood xylan (Figure 4A and B). In both cases the release of X3, X4, and X5 was observed after 15 min of exposure to the enzyme, and increased levels of xylooligomers were observed at up to 60 min of incubation. After 16 h the levels of xylooligomers decreased and the presence of xylose (X1) was detected. Xylose release was also observed in the xylan from oat spelt after 16 h; however, the hydrolysis profiles after 15 min, 60 min, and 16 h were significantly distinct (Figure 4C), which is a consequence of the different composition of this substrate in comparison with birch and beechwood xylans. Oat spelt xylan contains arabinoses as substituents of the xylose main chain, whereas birch and beechwood xylans present mainly glucuronic acid substituents, fewer arabinose ramifications, and extensive acetylation of the main chain xylose residues [24, 25]. The results obtained suggest that MpXyn10A also has a good activity against xylan polymers that contain acetyl group modification on the main chain xylose sugars, as reported in Table 2.

The catalytic activity of MpXyn10A was also measured with xylan from birchwood and oat spelt using the Miller method (Table 2). The purified recombinant enzyme presented activity against oat spelt and birchwood in close levels, but the crude extract from the wild-type *M. pulchella* presented a higher specific activity against oat spelt than birchwood.

### Sugarcane bagasse hydrolysis

The use of sugarcane bagasse for bioethanol production requires pretreatment to improve access of enzymes and digestibility by removing core and non-core lignin fractions. Many physical and chemical methods can be employed for biomass pretreatment, including steam explosion, gamma radiation, and treatment with alkali, hydrogen peroxide, and solvents, among others [26]. These treatments are used in order to increase the inner surface area of the substrate and open the cellulose fibers to improve solubilization and degradability of hemicellulose and lignin [27].

**Figure 5:**
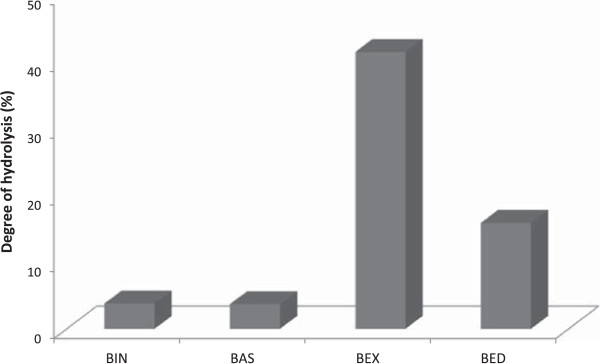
**Enzymatic hydrolysis of sugarcane bagasse.** MpXyn10A was incubated with bagasse *in natura* and differently pretreated sugarcane bagasses for 5 h in 50 mM sodium acetate buffer, pH 5.5, at 55°C. The amount of reducing sugars released was quantified as previously described by Miller [33]. BIN - bagasse *in natura*, BAS - bagasse pretreated with sulfuric acid, BEX - bagasse exploded, and BED - bagasse exploded and delignified.

The results of the hydrolysis of the sugarcane bagasse after different pretreatments using the purified recombinant MpXyn10A are presented in Figure 5. After incubation with MpXyn10A, experiments with all the different bagasses presented an increased amount of reducing sugar, even for the bagasse *in natura* (BIN)*.* A higher hydrolysis occurred in the steam exploded bagasse (BEX), with about 41.5% of hemicellulose hydrolysis, followed by exploded and delignified bagasse (BED,15.9%), BIN (3.8%), and sulfuric acid-pretreated bagasse (BAS,3.7%). BEX presented the most promising result, releasing an amount of reducing sugars about 10 times higher than that released by sugarcane BIN. It is important to appreciate the differences between each of the sugarcane bagasses used in this experiment, since each pretreatment introduces chemical changes in the biomass composition and structure.

The composition of each bagasse is presented in Table 4 [28]. Steam explosion exposes the available hemicellulose, allowing access to and hydrolysis by MpXyn10A. However, when steam explosion is followed by a delignification step, the treatment with NaOH also removes a significant fraction of the hemicellulose from the bagasse, reducing the percentage of hemicellulose in BED. The reduced hemicellulose content has a negative impact on the hydrolysis of this material, resulting in the low yield of reducing sugars on treatment with the MpXyn10A (Figure 5). The pretreatment with sulfuric acid changes the content of hemicelluloses but not the content or arrangement of the lignin in the fibers, which may be responsible for blocking the enzyme from acting in the bagasse fibers. Consequently, the percentage of sugar release was very close to that observed using BIN (3.7% with BAS compared to 3.8% with BIN). It is noteworthy that previous reports using the xylanase from *Thermomyces lanuginosus* [21] reported a maximum of 2.6% hydrolysis using sugarcane bagasse submitted to thermal (steam explosion) pretreatment, a value much lower than the 41.5% observed in the current study.

**Table 4:** **Composition of the bagasses** [28]** tested for MpXyn10A biomass degradation**

Composition	BIN (% of mass)	BAS (% of mass)	BED (% of mass)	BEX (% of mass)
Cellulose	43.8 ± 1.1	58.4 ± 1.4	89.5 ± 1.6	51.7 ± 0.6
Hemicellulose	25.8 ± 0.8	6.5 ± 0.3	3.4 ± 0.3	8.9 ± 0.1
Lignin	22.1 ± 0.8	32.0 ± 0.2	5.5 ± 0.2	34.3 ± 0.3
Others	8.0 ± 1.2	3.2 ± 0.2	2.6 ± 0.2	5.1 ± 0.2

Others correspond to ash and extractives.

The results obtained in this work with the recombinant MpXyn10A show potential for use of this enzyme in biomass degradation. Characteristics such as its high thermostability allow the MpXyn10A to act on the substrate for long periods, allowing a more complete hydrolysis of the substrate. In addition, the results with the pretreated bagasses showed an increase in reducing sugar release, and the increase in released fermentable sugars could be further used by yeasts to produce second generation ethanol.

## Conclusions

A thermostable endoxylanase from *M. pulchella* was expressed as a heterologous protein in *Aspergillus nidulans*, and the biochemical characteristics of the enzyme show potential for biotechnological applications, such as second generation ethanol production. The high thermostability of the enzyme allows it to act on the substrate for prolonged periods, which presents clear advantages for the production of higher amounts of fermentable sugars from pretreated sugar cane bagasse.

## Methods

### Microbial strains, plasmids, and culture conditions

*Malbranchea pulchella* was cultured in a defined minimal medium (MM) [29] supplemented with 1% glucose and 0.1% yeast extract at 45°C at 180 rpm. *A. nidulans* was cultured in complete medium (CM) as previously described [30]. One Shot® TOP10 Chemically Competent *E. coli* (Invitrogen) was used to propagate plasmids and to clone purified polymerase chain reaction (PCR) products. The PyrG-containing vector pEXPYR was used for the expression of MpXyn10A in *A. nidulans* [15].

### Cloning of MpXyn10A

*Malbranchea pulchella* mycelia were harvested and frozen with liquid nitrogen, and genomic DNA was prepared by treatment with 600 μL of genomic extraction solution (50 mM EDTA, 1% SDS). The mycelium suspension was heated at 68°C for 10 min and centrifuged at 13,000 *g* for 5 min, and the supernatant was transferred into a new tube. A volume of 40 μL of 5 M potassium acetate was added, mixed by inversion, placed on ice for 10 min, centrifuged at 13,000 *g* for 5 min, and the supernatant transferred to a new tube containing 2.5 volumes of 95% EtOH. The solution was centrifuged, and the DNA pellet was washed twice in 70% EtOH, air dried, and resuspended in DNAse-free water [31]. The ORF for the gene *Mpxyn10A* was PCR amplified with Pfu DNA polymerase (Thermo Scientific) using forward and reverse primers designed from a multiple *xyn10* sequence alignment of sequences extracted from GenBank [GenBank:KJ767674]. The primer sequences were 5_NNNNN**CCTCAGC**AATGGTCGGCTTCTGCAGTTTTG 3 _ (forward) and 5_NNNNN**TCTAGA**TCACAAGCACTGCCAGTACCAATCATTCAG 3 _ (reverse), including restriction sites for directional cloning using *BbvC*I and *Xba*I sites (indicated in bold). The following touchdown PCR cycle parameters were used: denaturing at 95°C for 1 min, annealing at 60°C for 30 s, and extension at 68°C for 3 min, with cycle repetition in which the annealing temperature was reduced by 2°C every cycle until an annealing temperature of 52°C was reached, then 28 cycles of 95°C for 30 s, 52°C for 30 s, and 68°C for 3 min, followed by a final extension at 68°C for 10 min [15].

### Heterologous expression, secretion, and MpXyn10A purification

The PCR product was ligated in-frame and in-line to *BbvC*I and *Xba*I restriction sites of the high-yield expression and protein secretion vector pEXPYR [15, 31]. The cloning junctions and entire MpXyn10A insert (including the CBM1 and catalytic domains) were sequenced and transformed [32] into *A. nidulans* strain A773 (*pyrG89, wA3, pyroA1*). Transformants were isolated by their ability to grow in the absence of uracil and uridine. Protein expression was carried out in Erlenmeyer flasks using MM [29] with 5% maltose as inducer at 37°C under static conditions. MpXyn10A production was monitored by analyzing the culture supernatant using SDS-PAGE and the hydrolytic activity on birchwood xylan (Sigma) as measured by reducing sugar production measured by the dinitrosalicyclic acid (DNS) method [33].

The crude extract obtained from 5% maltose induction culture was purified by Biogel P-60 (1.0 cm × 125.0 cm) equilibrated in 50 mM sodium acetate buffer pH 5.5. The column was eluted in 1-mL fractions at a flow rate of 0.1 mL/min. A volume of 10 μL from each fraction was used for SDS-PAGE and for activity assays. The fractions that presented xylanase activity and showed a single band on SDS-PAGE were collected and pooled.

### Enzyme characterization

Enzymatic activity was measured by colorimetric assay using birchwood xylan as substrate, and the reducing sugars were determined according to the Miller procedure [33] using xylose as control. The reaction mixture (0.05 mL substrate (1% w/v) in 50 mM sodium acetate buffer, pH 5.0, and 0.01 mL enzyme solution) was incubated at 70°C in a water bath for 5 min. The reaction was stopped by adding 0.1 mL of DNS and immediately boiling for 5 min. Quantification of the reducing sugars released as a result of enzyme activity was estimated by A_540_ measurements, where one unit of enzymatic activity was defined as the amount of enzyme which produced 1 μmol.min^-1^ of reducing sugars.

In order to determine the optimum pH and temperature profiles, the enzymatic reaction was carried out at different pH values in citrate-phosphate buffer (pH 3.5 to 8.0) and various temperatures (45 to 90°C). In order to determine the thermostability, MpXyn10A was incubated at 65, 70, or 80°C for up to 24 h. Samples were taken at different times, and the catalytic activity was measured. The assay for beta-xylosidase activity was carried out with 0.5% p-nitrophenyl-beta-D-xylopyranoside in 100 mM sodium succinate buffer, pH 5.0. The reaction was stopped by the addition of saturated sodium tetraborate, and the absorbance was measured at 420 nm. The protein content was measured by the Bradford method [34]. The assays were performed in triplicate in at least three independent experiments.

### Zymogram gel

In order to perform the zymogram analysis, the enzyme was electrophoresed as 12% SDS-PAGE without addition of beta-mercaptoethanol and without boiling of the sample. After running, the gel was washed five times for 20 min with 50 mM acetate buffer, pH 5.5. The gel was then incubated for 30 min at 80°C in the same buffer containing 0.5% xylan before soaking in 0.1% Congo Red solution for 30 min, at room temperature, and posterior washing with 1 M NaCl until the excess dye was removed from the gel [35].

### Determination of K_M_ and V_max_ of MpXyn10A

In order to determine K_M_ and V_max_ of MpXyn10A, the birchwood xylan substrate was used over a concentration range from 1.125 to 18 mg/mL in 50 mM sodium acetate, pH 5.5, and the activities were determined under standard conditions. The software SigrafW [36] was used to calculate the kinetic parameters.

### Capillary zone electrophoresis of oligosaccharides

The oligosaccharides released by the enzyme action on the Xyn10A were derivatized with 8-aminopyrene-1,3,6-trisulfonic acid (APTS) by reductive amination as described previously [37]. Enzymatic reactions were performed as described for standard enzymatic activity assays using birchwood, beechwood, and oat spelt xylan, and incubation at 65°C for up to 16 h. Capillary zone electrophoresis of xylohexose breakdown products was performed on a P/ACE MQD system (Beckman Coulter) with laser-induced fluorescence detection. A fused-silica capillary (TSP050375, Polymicro Technologies) of 50-μM internal diameter and 31-cm length was used as the separation column for the oligosaccharides. Samples were injected by application of 0.5-psi pressure for 0.5 s. The electrophoresis conditions were 10 kV/70 μA with the cathode at the inlet, 0.1 M sodium phosphate (pH 2.5) as running buffer, and a controlled temperature of 20°C. The capillary was rinsed with 1 M NaOH followed by running buffer to prevent carryover between runs. The APTS-labeled oligomers were excited at 488 nm, and emission was collected through a 520-nm bandpass filter. Because of the small volumes of the capillary, the electrophoresis combined small variations in buffer strength, and retention times varied slightly when comparing separate electrophoresis runs. The combined information obtained from the electrophoretic behavior and coelectrophoresis with xylan oligosaccharides was used for the identification of hydrolysis products.

### Differential scanning fluorimetry

Differential scanning fluorimetry was used to determine the melting temperature of XynA, glycosylated and deglycosylated. Stability measurements were performed using an Agilent Mx3000P QPCR machine and the fluorescent dye SYPRO Orange (Sigma-Aldrich) diluted 2,000-fold from the stock solution [37]. The fluorescence was measured with excitation and emission wavelengths of 517 and 585 nm, respectively. All experiments were performed in 20 mM sodium acetate buffer, pH 5.5, with protein at 25 μM, and a total volume of 30 μL. The fluorescence was monitored while increasing the temperature in steps of 1°C at 30-s intervals from 25 to 96°C. The melting temperatures (Tm) were calculated by fitting a sigmoidal curve to the data using the MTSA46 program for MATLAB [38].

### Molecular modeling

A PSI-BLAST (Position-Specific Iterated Basic Local Alignment Search Tool) [39] search with default parameters was performed against the Protein Data Bank (PDB), and GH10 xylanase from *Fusarium oxysporum* (PDB code 3U7B [5]) and a cellulose-binding domain (CBD) of endoglucanase I from *Trichoderma reesei* (PDB code 4BMF [20]) were selected for building a structural model of the XYN10A by comparative modeling techniques with the program MODELLER9v12 [40]. The structural model was validated utilizing the program PROCHECK [41], which showed that 91% of the residues were in highly favored regions of the Ramachandran graph. The prediction of N- and O-glycosylation sites was estimated by NetNGlyc1.0 and NetOGlyc3.1, respectively.

### Circular dichroism

The far-ultraviolet circular dichroism (CD) spectra of xylanase were measured between 190 and 260 nm in 10 mM phosphate buffer, pH 5.0, at 25°C with a J-810 spectropolarimeter (Jasco, Hachioji City, Tokyo, Japan) flushed with nitrogen gas using 2-mm path-length cuvettes and a protein concentration of 0.1 mg/mL. For each measurement, a total of six spectra were collected, averaged, and corrected by subtraction of a buffer blank. The percentage of second structure from the CD spectra was estimated according to Raussens’ method [42].

### Avicel and oat spelt xylan binding assay

The binding assay was conducted using 10 mg of microcrystalline cellulose (Avicel) or oat spelt xylan. The polysaccharides were washed and equilibrated with 50 mM sodium acetate buffer, pH 5.5, and were incubated with 80 μg of either the glycosylated or deglycosylated MpXyn10A for 30 min at 4°C, under agitation. The mixtures were then centrifuged at 13,000 *g* for 5 min at 4°C. The supernatant was used for enzymatic assay and SDS-PAGE, and the polysaccharides were washed five times with the same buffer and centrifuged at 13,000 *g* for 5 min each time. The enzyme bound to the polysaccharides was assayed for activity by adding 0.5% birchwood xylan and incubation at 70°C for 10 min. After centrifugation at 13,000 *g* for 5 min, DNS was added to the supernatant, the mixture was boiled for 5 min, and the absorbance was read at 540 nm.

### Hydrolysis of sugarcane bagasse with different pretreatments

The hydrolysis was conducted in sugarcane bagasse with bagasse *in natura* (BIN) and three pretreatment bagasses: acidic treatment (BAS), exploded and delignified bagasse (BED), and exploded bagasse (BEX) [28]. All the biomasses used were washed thoroughly with 50 mM sodium acetate buffer, pH 5.5, to remove residual soluble sugars. A 1.5% w/v suspension of the treated and washed substrate was prepared in the same buffer and mixed with 0.5 nmol of purified enzyme in a final reaction volume of 1.5 mL. The reaction was incubated at 55°C for 5 h in a homogenizer pump to avoid substrate precipitation, and the total reducing sugar release was measured by the DNS method as described above. The temperature of 55°C was chosen in order to test the application of MpXyn10A in a biorefinery context, where high temperatures would not be desirable due to increased process costs.

Since the composition of the various bagasses differed, the percentage of hemicellulose hydrolyzed in each one was calculated. For this calculation, it was assumed that all the released reducing sugar was xylose, and the following equation was used:


The percentages of hemicelluloses in the specific bagasses are given in Table 4.

## Abbreviations

A773: strain of *Aspergillus nidulans* used in this work (*pyrG89, wA3, pyroA4, veA1*)
BAS: sugarcane bagasse pretreated with sulfuric acid
BED: sugarcane bagasse pretreated with steam explosion followed by alkali treatment for delignification
BEX: sugarcane bagasse pretreated with steam explosion
BIN: sugarcane bagasse *in natura*
CBM: cellulose-binding domain
ESI-MS: electrospray ionization mass spectrometry
far-UV CD: far-ultraviolet circular dichroism
GH10: glycosyl hydrolase family 10
kcat: turnover number
KM: Michaelis constant
LC-MS: liquid chromatography-mass spectrometry
MpXyn10A: xylanase of *Malbranchea pulchella* expressed in *Aspergillus nidulans*
ORF: open reading frame
PDB: Protein Data Base
pEXPYR: vector used in this work
pNP: ρ-nitrophenol
Tm: melting temperature
Vmax: maximum reaction rate
X1: xylose
X2: xylobiose
X3: xylotriose
X4: xylotetrose.
